# Efficient light harvesting using simple porphyrin-oxide perovskite system

**DOI:** 10.1038/s41598-020-70554-5

**Published:** 2020-08-24

**Authors:** Shalu Sharma, Sandeep Chhoker

**Affiliations:** Department of Physics and Material Science and Engineering, Jaypee Institute of Information Technology, Noida, Gautam Buddha Nagar, India 201307

**Keywords:** Solar cells, Electronic devices

## Abstract

Here, we report the systematic studies on photoanodes of phase pure polycrystalline microrods of Barium Stannate (BaSnO_3_) microrods for application in porphyrin dye-sensitized solar cell (DSSC). We were able to establish the effect of vacuum annealing on BaSnO_3_ thin films on its electrical, optical and adsorption properties using XPS, UV–Vis, photoluminescence and adsorption isotherm studies. Increase in oxygen vacancy with annealing is found to increase the room temperature (RT) electron mobility from 49.1 to 82.4 cm^2^/V sec whereas macroporous nature of samples were found suitable for faster dye adsorption (~ 30 min). Post TiCl_4_ treatment studies, the maximum efficiency (η) of 4.7% is achieved in BSO films with current density J_sc_ ~ value as 10.4 mA/cm^2^; whereas DSSC fabricated using annealed BSO films gave maximum efficiency of 6.1% with J_sc_ value as 12.2 mA/cm^2^, during which the value of FF increased from 73.4 to 81%. The IPCE and proposed electron transfer mechanism suggested the potential application of macroporous BSO with unconventional dyes such as metallised-porphyrin. Our results strengthen the idea of using phase-pure, visible transparent porous BSO nanostructures with induced oxygen vacancies due to annealing process post-synthesis which eventually increased DSSC performance from by 84%.

## Introduction

Visible range transparent photoanode based solar cells^1^ called as DSSCs have attracted much attention recently owing to its ease of fabrication as well as it being inexpensive and environmentally friendly at the same time. Some of the key areas for research in DSSCs have been the improvement in interfacial charge recombination^2,3^, metal-ion dye complex formation^4^, dye absorption durations^5^, electron mobility^6^, phase purity^7,8^, synthesis strategies^9,10^. These areas were more so explored because of pertaining limitations to presently used photo-anodes based on simple binary oxides such as ZnO and TiO_2_ as well as the complex ternary oxide such as BaSnO_3,_ Zn_2_SnO_4_, SrTiO_3_, SrSnO_3_ and BiFeO_3_^11,12^. Importantly these ternary oxides provide more freedom to modify their chemical properties and band structures as compared to surface modification of binary oxides. However, the surface modifications of ternary oxide photoanodes such as Zn_2_SnO_4_, SrSnO_3_, and BaSnO_3_ have not been investigated in detail. Zn_2_SnO_4_ has already drawn substantial interest due to lower electron–tri-iodide recombination rate^13,14^. In our previous study, we carried out work to understand behaviour of Zn_2_SnO_4_ nanowires-based photo-anode thin films for its suitability with unconventional porphyrin dye for which the IPCE comes out to be ~ 28%^15^.

However, much attention has been paid to a very interesting perovskite oxide(both in bulk as well as thin film form); barium stannate (BaSnO_3_, BSO; E_g_ ~ 3.2 eV) for its high electron mobility (320 cm^2^/V-Sec in La-doped thin films), faster dye absorption rate, n-type resistivity (10^–2^–10^–4^ Ω cm) and low visible absorption^16,17^. Recently, there have been many reports on BSO based photoanodes with an overall efficiency from 1.1^18^ to 6.2% with TiCl_4_ treatment^5,19^. Besides this, there were reports on doped BSO^20,21^, morphology and film thickness optimization studies^22,23^ for its improved performances. Recently Roy et al.^5^. reported an interesting study on porous nanorods of BSO and its dye absorption capabilities, using dextran as template for DSSC studies. Earlier, there were fewer reports on synthesizing porous BSO without templates in which BaCO_3_@SnO_2_ core shell structures were calcinated to get 1D-porous BSO nanorods^24^. However, phase purity and conversion of carbonate phase to stannate phase remains an issue to address, ensuing its low conversion efficiency. Herein, Co-precipitation type of synthesis requires precise pH control in order to regulate cations and anions in solutions and results in formation of self-assembled structures as we achieved in this study. Thus, our interest was to use these porous structures of BSO and study its dye absorption performance with unconventional dyes (non-poisonous macrocyclic compounds like Porphyrin, Pthalocyanine and Corroles). Porphyrin as photo-sensitizers was particularly of interest owing to its role in light harvesting in photosynthesis process^25^. In recent years, research into porphyrin dyes, in particular the push–pull type dipolar Zn(II) porphyrins, for DSSCs have tremendously increased because of its intense absorption in wavelength region of 400–480 nm (Soret band, extinction coefficient ε > 110,000 /M-cm ) and 500–700 nm (Q-band, ε > 20,000/M cm), the appropriate LUMO and HOMO energy levels^25–27^ with versatile structures such as YD-2 porphyrin reported by Gratzel et al.^26,27^ with PCE as high as 11% and PCE as high as 13%^28^ with TiO_2_ transport layer wherein its application is not limited to substitute ruthenium dyes but in many other visible range applications also. There are several studies which emphasises the challenges in harvesting the sunlight for the entire spectral region for efficient injection of photoexcited electrons into photoanodes using only a single porphyrin dye sensitizer and thus, co-sensitized methods have also been reported in 2011, with PCE of 12.3% for DSSCs^29^. However, the J–V stability in most the reports have not been carried out. Such porphyrin dyes are required very recently for its ensuing application in DSSC, as compared to that of Ru-based sensitizers. The advantages of choosing porphyrin as an alternative for Ru-based complexes are their very high molar extinction coefficients, free from expensive and toxic Ru metal, highly tuneable absorption energies, stability under alleviated temperature and prolonged illumination besides its facile synthesis. On the similar lines, there is a rapid progress towards perovskite solar cells wherein various type of sensitizer’s e.g. Ru-based, macrocyclic compounds like porphyrins, phthalocyanines, different electron transporting layer materials like Perovskite organo-halide (CH_3_NH_3_PbX_3_) based light harvesters^30^ have been used for simpler interfacial charge recombination. Through this study, the strategy is proposed for synthesizing porous microrods of BSO and further using it with a donor type porphyrin dye, without using TiO_2_-type scattering layer, and further possibility of increasing surface area/porosity via TiCl_4_ treatment, which could open studies in hybrid solar cell. Although long term stability is still a vital issue before any commercialization of DSSCs but the possibilities with BaSnO_3_ micro/nanostructures for its phase pure synthesis, porous structures and using it with porphyrin dye will be a major contribution through this work. The choice of porphyin dye is also based on its long life time in its excited singlet state (> 1 ns), very fast electron injection rate (in femto-second range) and milli-second time scale electron recombination rate. In this study, we carried out the systematic studies on pH dependent synthesis of porous microrods of BSO and further its structural, optical and electrical properties for its performance as photo-anode using porphyrin dyes as a substitute to toxic Ru-based sensitizers.

## Methods

### BSO micro-rods synthesis

Powder of BaSnO_3_ was prepared by low temperature co-precipitation method using pure 20 mM BaCl_2_.2H_2_O (Merck, India) and SnCl_4_.5H_2_O (Merck, India) precursors in 0.2 M solution. 25% NaOH (Merck, India) was used as pH controller. Initially aqueous solution of BaCl_2_.2H_2_O was taken which was simultaneously heated and stirred until temperature reached 75 °C. As the pH value reached above 9, with NaOH addition, aqueous solution of SnCl_4_·5H_2_O was added and the yellow precipitates formed immediately. It is important to mention that precipitate was white as the pH value reached 11.5 in above process. It is assumed that a solution should have pH, high enough to exceed the BaSnO_3_ solubility and cause its precipitation and thus excess NaOH was added. The solution was then allowed to remain at 95 °C for 1 h and was further dried in oven for 24 h at 400 °C. The prepared powders were then calcinated at 1,100 °C in furnace for 4 h. The obtained yellowish-white powder is called as BSO throughout the manuscript. The obtained precipitate is barium tin hydroxide, which converts to perovskite structured BaSnO_3_ at above 400 °C for 4 h. Possible reaction equation is given below:

$$ \begin{aligned} {\text{BaCl}}_{2} \cdot 2{\text{H}}_{2} {\text{O}} + {\text{SnCl}}_{4} \cdot 5{\text{H}}_{2} {\text{O}} + 6{\text{NaOH}} & \to {\text{BaSn}}\left( {{\text{OH}}} \right)_{6} \downarrow + {\text{6NaCl}} + {\text{7H}}_{{2}} {\text{O}} \\ {\text{BaSn}}\left( {{\text{OH}}} \right)_{6} &  \xrightarrow{\Delta } {\text{BaSnO}}_{3} + 3{\text{H}}_{2} {\text{O}} \\ \end{aligned} $$

Previously^31,32^, similar reactions have been utilized to synthesize acicular BaSnO_3_ crystals through intermediate phase of BaSn(OH)_6_, however pH responsive segregation as well as the synthesis of porous microrods wherein calcinations duration and temperature is comparatively lower as compared to previous reports, have been achieved in this study. Reason for this is assumed to be higher pH values based segregation due to ions de-localisation in BaSn(OH)_6_ matrix.

### Characterization details

The crystallographic phases of all the samples were determined by powder X-ray diffraction (XRD) study using XRD-6000 Shimadzu analytical diffractometer with CuK_α_ (λ = 1.54 Å) radiation. The specific surface area was measured using Autosorb iQ2 and specific surface area were calculated using BET (Brunauer–Emmett–Teller) method whereas pore size distribution is measured using BJH (Barrett-Joyner-Halenda) method using liquid nitrogen physisorption isotherm measured at 77.4 K. The optical properties have been investigated by diffuse reflectance spectroscopy using Ocean optics UV–visible 4,000 spectrophotometer. The FTIR spectrum has been measured between 4,000 and 400 cm^−1^ using Perkin-Elmer spectrum-BXII. Field Emission Scanning Electron Microscope FEI Quanta 200F with Oxford-EDS system (operated between 5 and 30 kV) were used for morphological studies. Micro Raman spectrophotometer Renishaw, UK (samples were excited with the 514.5 nm line of Ar^+^ ion laser operating at 50 mW) was also used. Photo luminescence (Perkin Elmer-LS 55) was carried out to understand the defect states in the sample band gap. We performed chemical analysis of samples using X-ray Photoemission spectroscopic (XPS) instrument in a OMICRON Multiprobe Surface Analysis System operating at a base pressure of 5 × 10^−11^ Torr. All films were mounted on Mo plates using conductive tape to avoid charging. The XPS measurements were carried out by using Mg Kα (1,253.6 eV) radiation source. WE performed the Room temperature Hall coefficients measurements using Vander-Pauw probe methods from which RT electron mobilities, carrier concentrations, resistivity were calculated.

### Details of DSSCs

For DSSCs, thin films of BSO, BSO-A, BSO-T, BSO-AT were prepared on FTO glass (7 Ω/cm^2^) with area of ~ 0.7 cm^2^. Initially, Photoanodes are prepared by making paste of BSO and mixing α-teripenol and ethyl cellulose^15^. This paste was coated on FTO glass substrate using doctor blade technique. Further, these films were sintered at 300 °C, in multiple steps to eliminate any contribution from organic compounds used. After atmospheric cooling, these films were taken to vacuum furnace for annealing at 400 °C for 2 h and allowed to cool to room temperature. These films were then dipped in 1 mM ZnTPP dye in ethanol at room temperature for 15, 30 and 45 min. The amount of dye adsorbed on various BSO films was evaluated by UV–Vis and weighing anodes in a microbalance before and after the dye adsorption method and it is found that maximum dye loading occurs in about 30 min, after which dye loading gets saturated and thus we chose the BSO thin film in this study (thickness ~ 20 μm). We also performed this section of study with BSO samples annealed at 600 °C (BSO-A) but the dye loading amount was similar and also with no appreciable change in XPS results, except for oxygen element. It may be pointed here that, for other common photo-electrode materials the soaking period is large as compared to this process (e.g. 24 h for TiO_2_, 4 h for ZnO, 12 h for Zn_2_SnO_4_, 120 h for BiFeO_3_ etc.)^33–35^ as compared to BSO films. We also performed the TiCl_4_ treatment for comparison with BSO and BSO-A films. The pre-treatment of TiCl_4_ on BSO and BSO-A films was done by dipping FTO substrate in 0.05 M solution of TiCi_4_ for 15 min at 80 °C. These substrates were then further sintered at 400 °C for 30 min. After depositing BSO films over these substrates, post treatment of TiCl_4_ was performed by further dipping these BSO films for 10 min at 80 °C. Finally, these films were called as BSO-T (pristine BSO films with treatment of TiCl_4_) and BSO-AT (annealed BSO-A films with treatment of TiCl_4_). The idea of using TiCl_4_ is to increase the surface adhesion via efficient bonding between FTO substrate and semiconducting film for improved charge transfer from non-covalent linkage. Further, the working and counter electrode was sandwiched using a spacer to avoid short-circuit situation. Liquid iodide electrolyte (EL-HPE; Dyesol, Australia) was injected by drop between electrodes after which the electrodes were clipped together to maintain the planar cell geometry. Here, ~ 1 cm^2^ active area of the dye loaded films was used for the device performance. Thus, we carried out the J–V studies for BSO, BSO-A, BSO-T and BSO-AT films. The J–V characteristics of PV cell were measured using 1,000 W/m^2^ continuous one solar simulator and IQE-200 series system in 300–1,800 nm range was used for IPCE measurements.

## Results and discussion

**Figure 1:**
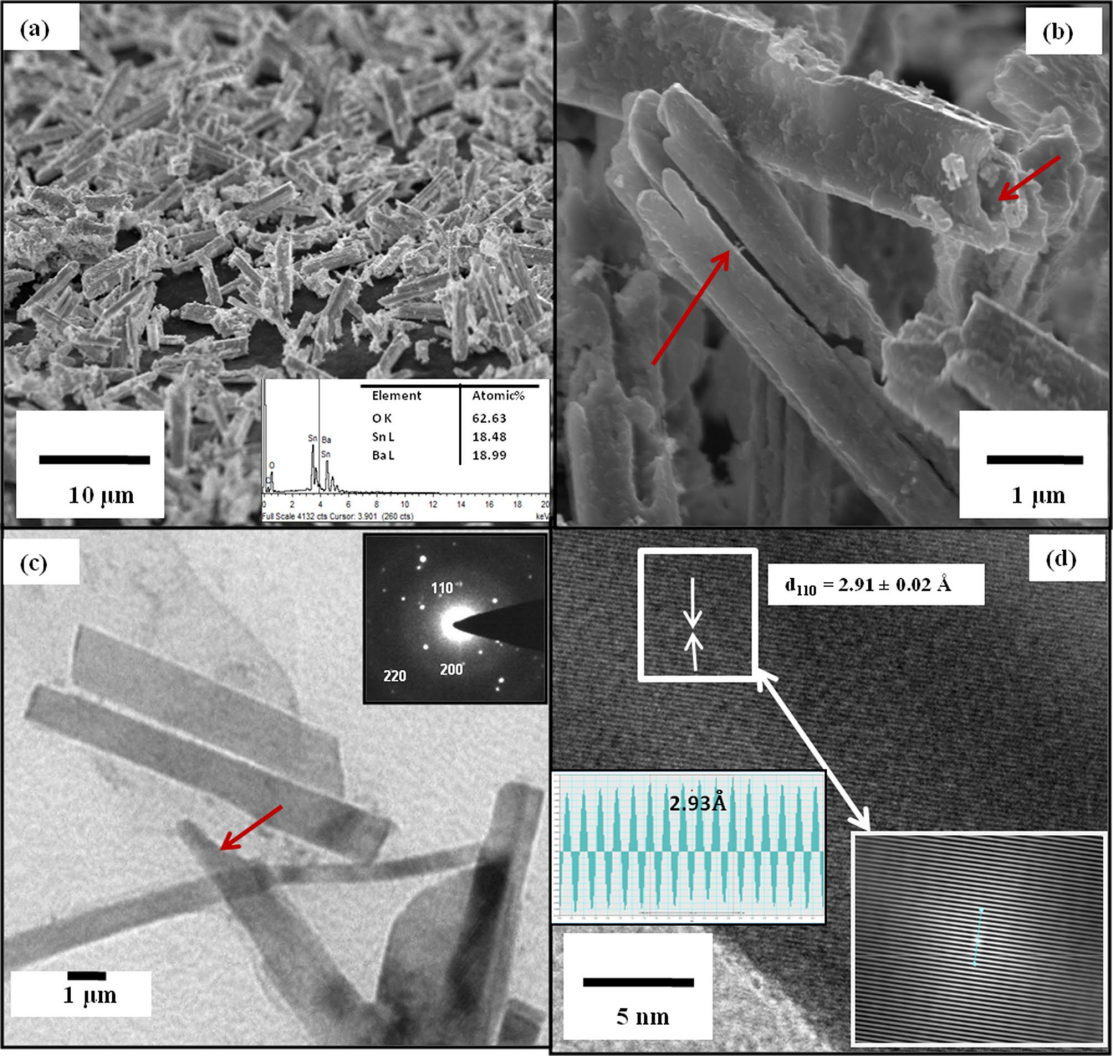
(**a**) FESEM image of BSO microrods; inset shows EDAX data corresponding to images (**b**) High resolution images of BSO microrods showing open ends (**c**) TEM bright field images; inset shows SAED pattern (**d**) HRTEM image; inset shows corresponding inverse fast Fourier transform pattern with lattice fringes.

Figure 1 shows the morphological studies of BSO samples synthesized wherein uniform porous micro-rods (Fig. 1a) were confirmed wherein polysaccharide support is not used. The EDX results (inset Fig. 1a) shows that the atomic % stoichiometry of Ba:Sn:O is 1:0.98:3.2 which well within the tolerance for ABO_3_ formation. The length of 10–15 μm and diameters 0.5–1 μm (Fig. 1b, c) for these rods were observed throughout the samples. These micro-rods were also seen with perforation as well as open edges (Fig. 1b, c which might help during surface dye adsorption). The micro-structures were found to be highly crystalline (Fig. 1d), in which most prominent inter-planar spacing of 2.9 Å is observed corresponding to (110) peaks; the growth direction of micro-rods apparently. Inset Fig. 1c shows the polycrystalline nature of sample in which (110), (200), and (220) planes were indexed, whereas inset Fig. 1d shows the FFT pattern of plane corresponding to the growth direction (110) as well as the lattice fringes calculations for (110) planes which comes out to be 2.91 Å with 0.02 Å accuracy.

**Figure 2:**
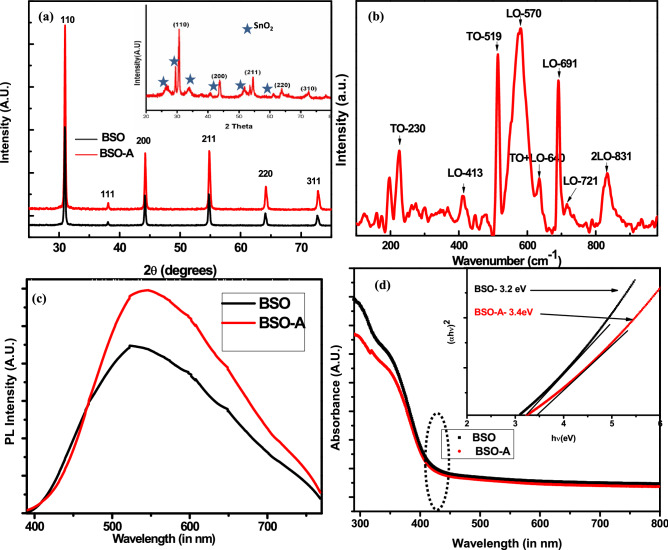
(**a**) X-ray diffraction pattern of BaSnO_3_ perovskite (BSO and BSO-A); inset shows impure BaSnO_3_ perovskite with SnO_2_ phase impurity (**b**) Raman spectrum (**c**) PL spectra of BSO and BSO-A samples (**d**) UV–Vis spectrum of BSO and BSO-A; inset shows corresponding Tauc’s plot.

Figure 2 shows the structural and optical properties of pristine BSO micro-rods. The corresponding XRD pattern of BSO sample confirms the pure BaSnO_3_ phase (inset Fig. 2a shows impurity SnO_2_ phase for synthesis at 9 pH value; as the solution goes more basic, formation of SnO_2_ is suppressed). Figure 2a shows the cubic BaSnO_3_ perovskite phase in which Reitveld fitting shows lattice parameter a = 4.108 Å for BSO and BSO-A samples. Figure 2a also shows the polycrystalline nature of sample in which (110), (111) (200), (211), (220) and (311) planes were indexed with average crystallite size of 43 nm (using Debye–Scherrer formula) and 47 nm with an ideal cubic perovskite space group *Pm3m* symmetry in BSO and BSO-A samples respectively. The Goldschmidt tolerance factor, defined by (r_A_ + r_O_)/√2(r_B_ + r_O_) is 1.01. The average crystallite size corresponding to inset XRD is ~ 50 nm. Considering the phase purity and crystalline nature of sample, further studies have been carried out for these BSO samples. Raman spectroscopy has been carried to further investigate the BaSnO_3_ formation and corresponding Raman spectra for pure BSO is provided in Fig. 2b (found same in BSO-A samples). Various longitudinal (LO) as well as transverse (TO) optical phonon modes at 230, 413, 519, 570, 639, 691, 721, 831 cm^−1^ have been identified, in which high intensity overtones were observed after 500 cm^−1^. The structure of BSO is characterized as a three-dimensional framework of SnO_6_ octahedra, which shares corner, corresponding to which the Sn–O-Sn bonding angle is 180°. Such types of bonding promote enhanced electron hopping between neighbouring Sn sites, which embodies the physical origin of high dispersion in conduction bands of BSO. No peaks corresponding to BaCO_3_ and locally disordered peaks due to Ba or Sn vacancy in crystal is observed. PL studies in Fig. 2c shows broadband emission in the visible spectral region called as green luminescence of synthesized BSO samples which follows inherent property of perovskite crystal family ABO_3_^36^. Though there is a shift from green emission to reddish-yellow in such morphology of BSO and the emission corresponding to 2.8 eV is from band edge emission. UV–Vis absorption of BSO samples (Fig. 2d) shows sharp increase in absorbance close to 390 nm in both pure as well as BSO-A samples and band gap calculated using Tauc’s relation is found to be ~ 3.2 eV in BSO and 3.4 eV in BSO-A samples. The increase in band gap here corresponds to oxygen vacancies being shallow donors and doesn’t introduce deep level in the band gap because of which only band edge emission^37^ is observed.

**Figure 3:**
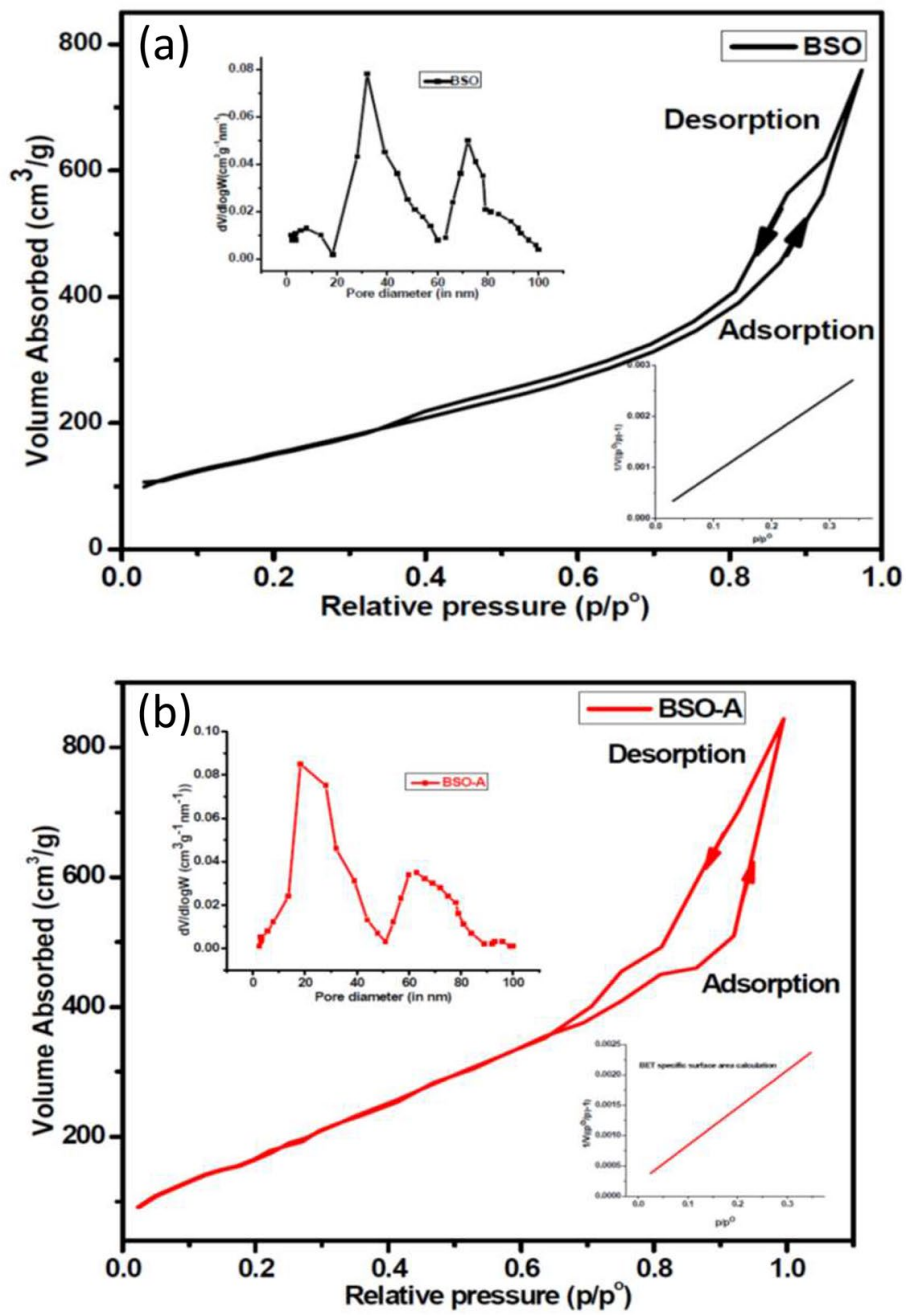
N_2_ adsorption/desorption isotherm (77 K) curves of (**a**) BSO and (**b**) BSO- A sample with specific area measurement fitting and porous volume/size distribution of the pore size (inset of each curve).

We performed the N_2_ adsorption–desorption measurements to ascertain information about the specific surface area and pore size distribution. The isotherms at 77.4 K are shown in Fig. 3. The isotherms of BSO and BSO-A samples are characteristic of a Type IV with type H2 hysteresis loop, which confirm the macroporous structure. The BET specific surface areas measurements (inset Fig. 3a, b down-right) and BJH pore measurement analysis were carried out from isotherms mentioned Fig. 3 and the corresponding results are shown in Table 1. It is evident from this study that suitable annealing temperature causes perforation in microrods because of which specific surface area as well as pore volume/size varies sufficiently to cause higher dye loading in BSO-A samples. The pore size distribution reveals a bimodal nature with a narrow distribution centered at 32 nm and a wide distribution centered at 73.4 nm in BSO sample. The BSO-A sample shows a narrow pore size centered at 19.5 nm and broader pore size distribution at 61.5 nm.

**Table 1:** Specific surface area and Pore size distribution results from BET-BJH calculations.

Sample name	BET specific surface area (m^2^/g)	Pore volume (cm^3^/g)	BJH pore distribution
BSO	574.3	0.17	Macroporous distribution of pores from 30–60 to 70–80 nm
BSO-A	687.2	0.21	Macroporous distribution of pores from 20–40 to 60–80 nm

**Figure 4:**
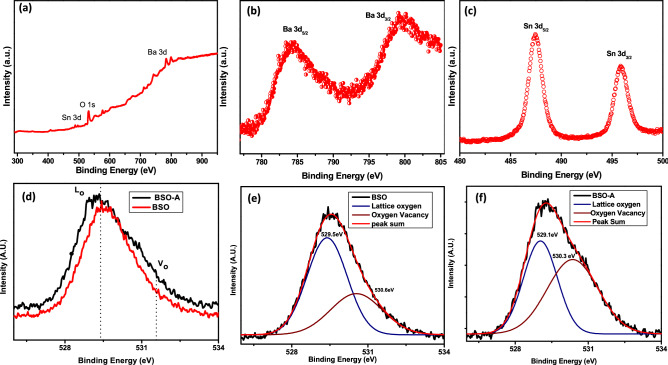
(**a**) XPS survey spectra and (**b**–**c**) Core level XPS spectra of Ba-3d and Sn-3d; (**d**) comparative Oxygen 1 s spectra of BSO and BSO-A films (**e**) deconvoluted O-1s spectra in BSO and (**f**) deconvoluted O-1s spectra in BSO-A films.

XPS analysis was carried out to ascertain the binding states as well as the chemical state of elements in pure BSO micro-rods and BSO-A and we didn’t observe any significant difference in these two samples except for O 1s spectra. The survey spectra is shown in Fig. 4a, whereas Fig. 4b, c shows the peaks of Ba 3d_3/2_ and 3d_5/2_ at 799.3 and 784.3 eV and Sn_3/2_ and Sn_5/2_ at 495.3 and 487.4 eV The XPS analysis confirms that the oxidation states of Ba (MNN), Sn (MNN) and O (KLL) were + 2, + 4 and -2 respectively. The possibility of any other metal–oxygen bond is prevented in this synthesis. It is to be pointed out here that the O 1s spectra are asymmetric towards higher binding energy (533–535 eV), thus indicating oxygen vacancies and is shown in Fig. 4d. This will eventually affect the electrical properties of any oxide-based semiconductor. Figure 4e, f shows the effect on oxygen vacancies as the BSO and BSO-A samples by deconvoluting the XPS plots mentioned in Fig. 4d. Increasing peak asymmetry towards higher binding energy indicates the effect of oxygen vacancy as compared to lattice oxygen. Peak area has been used to calculate V_o_/L_o_ ratio and is found to be 11% more in BSO-A samples; wherein L_o_ and V_o_ were the area under curve for lattice oxygen and oxygen vacancies in BSO samples^38^. The corresponding positions of lattice and vacant oxygen are found to be at 529.5 eV and 530.6 eV in BSO samples. Whereas, these positions are found to be at 529.1 eV and 530.3 eV respectively in BSO-A sample.

The room temperature electrical properties of BSO samples were carried out through Hall measurements (Table 2). The BSO micro rods were found to be n-type with resistivity and mobility of the order of ~ 10^–3^ Ω cm and 49.1 cm^2^/V s. In order to ascertain the annealing effect on the sample to study the change in resistivity and mobility and we found that there was increase in mobility (~ 82.4 cm^2^/V s.) in BSO-A samples which can be attributed to oxygen deficient samples although there is increase in carrier concentration (~ 3.2 × 10^20^ cm^−3^ as compared to ~ 9.4 × 10^19^ cm^−3^). These values of mobilities were higher than previously reported values of mobilities for pure thin films of BSO for which the reason is lesser grain boundaries, porous behaviour (ballistic transport) and longer conducting channels^38^ in macroporous BSO microrods.

**Table 2:** Summary of BSO RT electrical properties.

S. no	Sample	Mobility (cm^2^/V s)	Resistivity (Ω cm)	Charge carrier concentration (cm^−3^)
1	BSO	49.1	6.7 × 10^–3^	~ 9.4 × 10^19^
2	BSO-A	82.4	1.2 × 10^–3^	~ 3.2 × 10^20^

**Figure 5:**
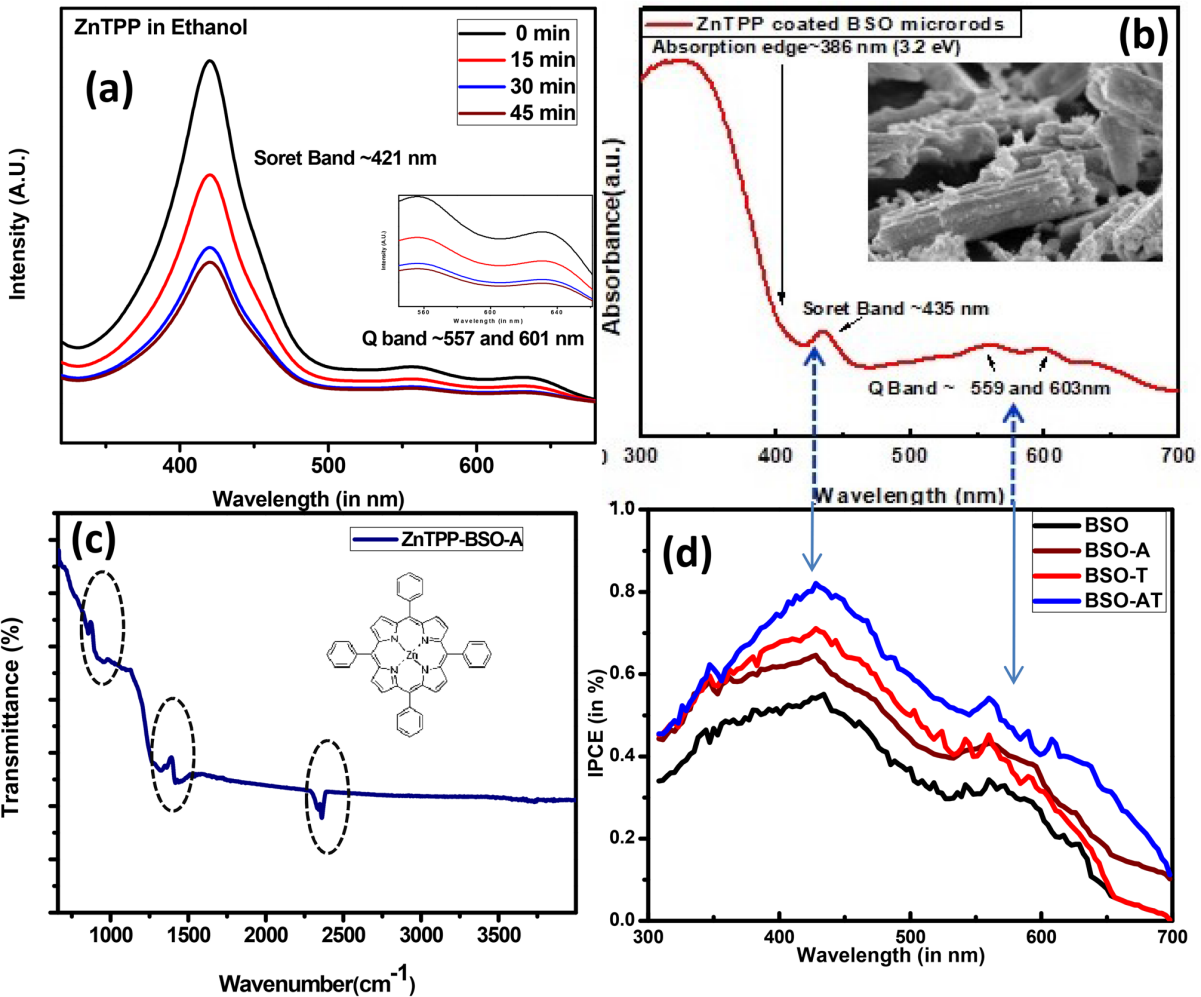
(**a**) Absorption spectra of ZnTPP in 1 mM ethanol solution for soaking period 0,15,30,45 min in BSO-A films (**b**) Absorption spectra of ZnTPP soaked BSO-A films (**c**) FTIR of ZnTPP soaked BSO-A films and corresponding chemical structure of ZnTPP dye and (**d**) IPCE of ZnTPP soaked BSO, BSO-A and BSO-T amd BSO-AT thin films.

We carried out the porphyrin dye adsorption of dye for 3 durations (15, 30, 45 min) at 1 mM in ethanol solution over BSO as well as BSO-A thin film for which optimization was carried out. The dye loading is understood by the corresponding UV–Vis plot of the pure solution (0 min) as compared to solution leftover in 15–45 min durations (Fig. 5a). We observed the ~ 50% intensity reduction in the solution from 0 to 30 min after which the % intensity change is not appreciable in 45 min solution (Fig. 4a) in both BSO as well as BSO-A. However, higher amount of dye loading is measured for thin films which were ~ 20 μm thick, the probable reason is large absorption coefficient of porphyrin, and for which the sample synthesis has already been discussed in experimental section. Figure 5a also shows the characteristic strong Soret band at 421 nm and Q-bands at 559 and 601 nm corresponding to Zinc tetraphenyl porphyrin (ZnTPP) in absorbance mode. Figure 5b shows the absorbance curve of ZnTPP modified BSO thin films in absorbance mode, in which absorption edge corresponding to BSO is observed at 3.2 eV (~ 386 nm) whereas Soret band and Q-band observed slight red shift attributed to weak π–π^*^ interaction between microrods and porphyrin molecules^39,40^. Figure 5c shows the FTIR spectrum of ZnTPP soaked BSO thin films in which signatures corresponding to BSO gets dominated by those of ZnTPP dye. Sharp bands at ~ 2,300 cm^−1^ and 1,300 cm^−1^ corresponds to ZnTPP dye whereas region of several peaks from 1,000 to 400 cm^−1^ belongs to BaSnO_3_. The peak positioning and intensities are also an indicative of stable interaction of dyes with BSO without any formation of new complex or aggregate. Thus, we performed IPCE measurements for four devices named as BSO, BSO-A, BSO-T and BSO-AT (Fig. 5d) wherein the role of TiCl_4_ in such DSSC’s has been identified to reduce surface recombination, bulk recombination or any loss in the electrolyte. We observed a broad spectrum in 300–700 nm range in all the cells with maximum value of ~ 81% at 435 nm corresponding to Soret band of porphyrin in BSO-AT, which is obviously higher than BSO (~ 55%), BSO-A (~ 62%) and BSO-T (~ 71%).

**Figure 6:**
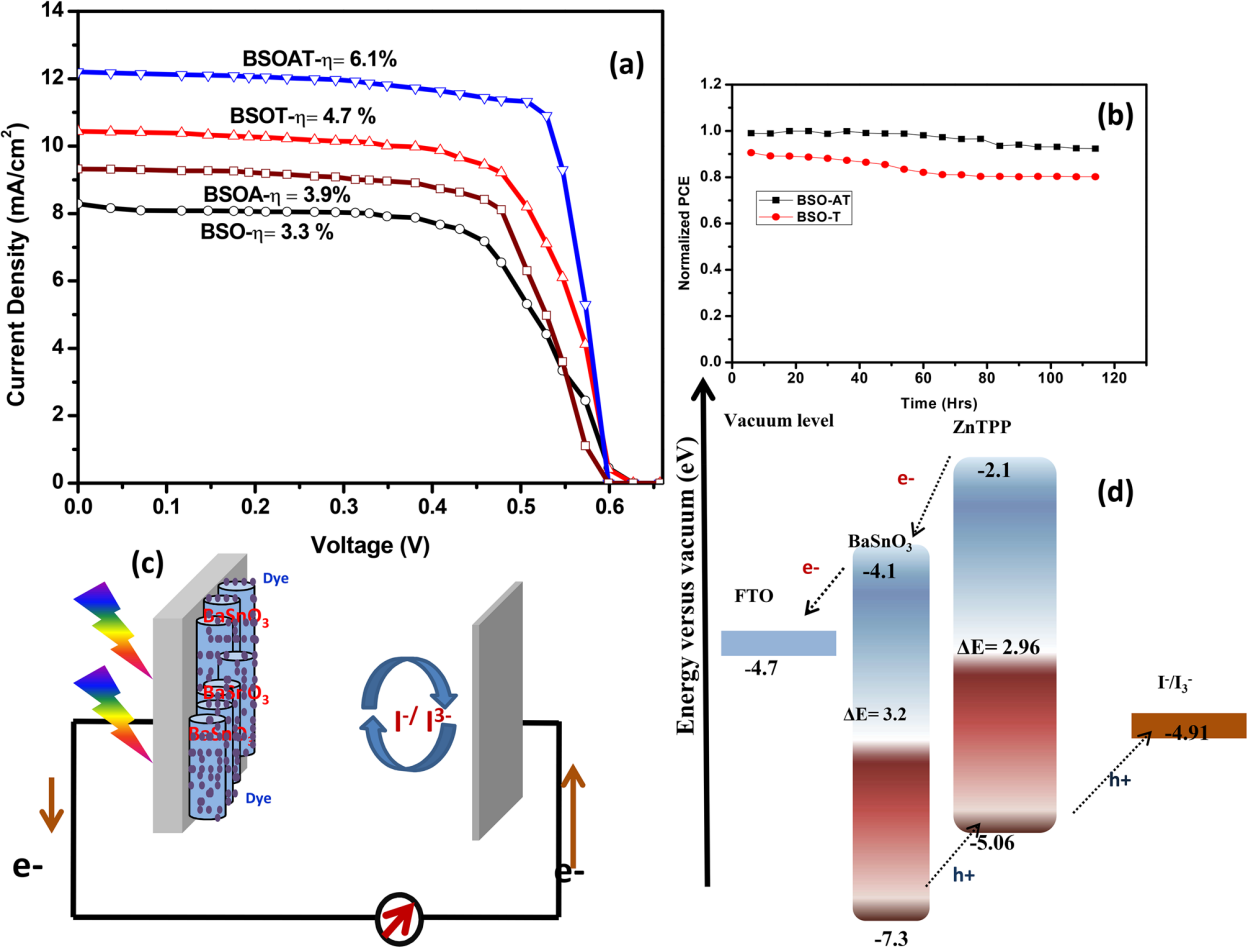
(**a**) J–V measurements of BSO, BSO-A, BSO-T and BSO-AT DSSC devices (**b**) Normalized PCE plot for BSO-AT device (**c**) Schematic of DSSC device used for measurement (**d**) Proposed electron–hole transfer mechanism and energy band diagram for the BSO-ZnTPP system.

The fabricated DSSC’s (schematic in Fig. 6c) were characterized by J–V measurements shown in Fig. 6a. A maximum efficiency of 6.1% was achieved for pure BSO-AT films which is major improvement for sporadic geometries of inorganic oxide micro rods morphologies sensitized with porphyrin dyes without using any scattering layer. The BSO thin films showed the maximum efficiency of 3.3% thus improvement by ~ 70% in BSO-AT based DSSC. The performance of all devices and its summary is provided in Table 3 and Normalized PCE stability plot is provided in Fig. 6b which shows cell stability within 10% range for 100 h. The DSSC devices exhibited highly competitive FF, V_oc_ and J_sc_ corresponding to previous reports on BSO thin films based DSSC and the comparison is provided in Table 4. The high values of V_oc_ and FF in BSO-AT is attributed to porous nature and increased surface area post annealing of BSO micro-rods (besides electrical properties reported in Table 2) which led to improved dye loading as compared to other geometries of BSO thin films based DSSC. Also, the annealing of BSO thin films increased the electron mobilities across substrate, which eventually played crucial role in enhancing its efficiency. Here, it is well known that porphyrin dyes relate to suppression of charge recombination between the electron injected into the conduction band of BaSnO_3_ and I^3−^ in the vicinity of BaSnO_3_. Some of the devices are compared in Table 4 for evaluating the performance of devices explored in this study.

**Table 3:** J–V performance of ZnTPP dye sensitized BSO DSSC devices.

S. no	Sample	J_sc_ (mA/cm^2^)	V_oc_ (V)	J_max_ (mA/cm^2^)	V_max_ (V)	FF (%)	η (%)
1	BSO	8.29	0.6	7.64	0.44	67.5	3.3
2	BSO-T	9.32	0.58	8.38	0.46	68.9	3.9
3	BSO-A	10.44	0.57	9.3	0.48	73.5	4.7
4	BSO-AT	12.2	0.6	11.2	0.53	81	6.1

**Table 4:** Reported values of DSSC devices using Barium Stannate and porphyrin/N719 dye.

#	Material type	BSO morphology	Sensitization duration	V_oc_ (V)	η (%)	FF (%)	Ref
1	Barium stannate + N719	Nanoparticles	24 h	0.68	1.1	60	13
2	Barium stannate + N719	Nanoparticles	–	0.6	5.2	66	16
3	Barium stannate + TiCl_4_ + N719	Nanoparticles	60 min	0.62	6.2	64	5
4	Barium stannate + TiCl_4_ + N719	Nanorods	20 min	0.8	6.86	54	4
5	Barium Stannate + TiCl_4_ + Porphyrin	Microrods	30 min	0.6	6.1	81	Present work

**Figure 7:**
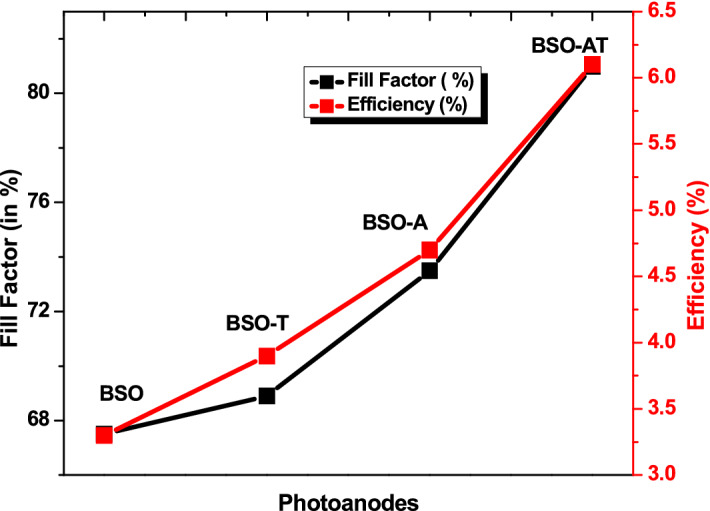
Comparative J–V for all four samples with different BSO photoanodes.

It is pointed here that metal oxide and dye-based electron–hole transfer system requires adequate band positioning including the one for ion conductor. In our case, the band diagram of DSSC system in Fig. 6d suggests that conduction band edge of BSO semiconducting layer is suitable to LUMO of porphyrin dye for electron transfer whereas the band positing for hole-transfer is more suitable for reduced electron–hole recombination. Thus, we can further improve this DSSC system by introducing another layer which makes conduction edge band even closer to LUMO of dyes for improved and efficient electron transfer. Porphyrin type dyes also helps in reducing dark current and surface recombination. Here the enhancement in J_Sc_ and V_oc_ indicates improved conduction path for electrons which in turn gets improved by TiCl_4_ treatment. A comparative chart for all samples J–V characteristics has also been added as Fig. 7. Overall, parameters like phase purity, crystalline, rod like shape, porosity, oxygen vacancies, high visible transparency, structural stability in different chemical environment, efficient response to TiCl_4_ treatment leads to this study in which porphyrin sensitized BSO DSSC are found suitable to progress in this field.

## Conclusion

The results presented in this article were quite interesting in many ways wherein the phase purity of BSO is achieved and annealed BSO thin films shows promising results after structural, optical and RT electrical studies. The evolution of macroporous microrods exhibited by BSO also plays important in dye loading capacity as well its soaking durations ~ 30 min. Maximum energy conversion efficiency of 4.7% was achieved for annealed BSO films which gets improved to 6.1% by post TiCl_4_ treatment. These results suggest the competitive nature of BaSnO_3_ perovskite as photoanodes wherein the low temperature phase pure synthesis porous barium stannate remains as the highlight of reported work. Although, there are several reports of higher efficiency with similar perovskite (6.86%) as well as sensitizing material (Porphyrin ~ 12%); we still emphasise the importance of this work on the basis of its simplicity, repeatability and tunability in both BaSnO_3_ (via doping of La, Al etc.); for even higher electron mobility and functionalized Porphyrin (YD-12, YD-13) for faster electron transfer mechanism and thus for higher efficiency.
